# Understanding implementation research collaborations from a co-creation lens: Recommendations for a path forward

**DOI:** 10.3389/frhs.2022.942658

**Published:** 2022-10-17

**Authors:** Mónica Pérez Jolles, Cathleen E. Willging, Nicole A. Stadnick, Erika L. Crable, Rebecca Lengnick-Hall, Jemma Hawkins, Gregory A. Aarons

**Affiliations:** ^1^ACCORDS Dissemination and Implementation Science Program, University of Colorado Anschutz Medical Campus, Aurora, CO, United States; ^2^Pacific Institute for Research and Evaluation—Southwest Center, Beltsville, MD, United States; ^3^Department of Psychiatry, University of California, San Diego, San Diego, CA, United States; ^4^Child and Adolescent Services Research Center, San Diego, CA, United States; ^5^University of California San Diego Altman Clinical and Translational Research Institute Dissemination and Implementation Science Center, La Jolla, CA, United States; ^6^The Brown School, Washington University in St. Louis, St. Louis, MO, United States; ^7^Centre for Development, Evaluation, Complexity and Implementation in Public Health Improvement, School of Social Sciences, Cardiff University, Cardiff, United Kingdom

**Keywords:** co-creation, principles, implementation, collaborations, CBPR

## Abstract

Increasing calls within the field of implementation science (IS) research seek to promote active engagement of diverse and often disenfranchised stakeholder voices to increase buy-in, fidelity, outcome relevance, and sustainment of evidence-based practices (EBPs). Including such voices requires cultural humility and the integration of multiple perspectives and values among organizations, groups, and individuals. However, the IS field lacks guidance for researchers on structuring collaborative approaches to promote a co-created process (i.e., synergistic approach to goal attainment). We contend that improved operationalization of co-created implementation collaborations is critical to sparking synergy and addressing differentials based on power, privilege, knowledge, and access to resources among stakeholders. These differentials can undermine future implementation and sustainment efforts if not addressed early in the research effort. An insufficient understanding of the guiding principles of co-created implementation collaborations may limit the scientific value of evaluation processes, and researchers' ability to replicate outcomes. We propose a perspective foregrounded in the concept of co-creation to guide the structuring of implementation collaboratives through five principles. We offer three case examples informed by the Exploration, Preparation, Implementation, Sustainment (EPIS) Framework to illustrate the application of these co-creation principles. Lastly, we offer recommendations for promoting co-creation in IS research moving forward.

## Introduction

Implementation strategies describe actions for promoting the uptake of evidence-based practices (EBPs), programs and policies (1). Implementation strategies often rely on multi-stakeholder collaborations to foster buy-in, inform implementation, and increase sustainment of EBPs (2). The Implementation Science (IS) field is re-assessing and broadening its approach to stakeholder engagement to incorporate the perspectives of a range of contributors, including patients, consumers, health professionals, and policy makers (hereafter called “stakeholders”) to tailor strategies to local contexts. Explicit concerns for involving individuals who experience health disparities, health injustices, and inequitable representation in the planning and implementation of EBPs and policies are increasingly central to such efforts.

Advancing health and social equity is critical to meeting IS goals of promoting action-based pragmatic research and closing the evidence-to-practice gap (3). Equity-centered IS entails naming researchers' assumptions, identifying our differences and systematic accountings of power relationships influencing study designs and decision-making (4). Equity-centered IS requires interaction with broader groups of stakeholders to identify, measure implementation strategies and outcomes, and to have an accurate understanding of how local contexts impact implementation (5–8).

We propose a perspective foregrounded in the concept of co-creation (i.e., synergistic approach to goal attainment) (9, 10), offering five principles to guide structuring implementation collaborations in research. Co-creation emerged in early late 1990s and early 2000s from business management (11) and has gained traction in healthcare and implementation research (12) as it has been heralded as a novel solution to the research to practice gap (13–15). We present three federally-funded implementation research studies as case examples to illustrate the application of these co-creation principles that are informed by the Exploration, Preparation, Implementation, Sustainment (EPIS) Framework (6). EPIS is a framework that guides the examination of determinants of implementation at the inner, outer and bridging levels and through four iterative phases as included in this name (16). Lastly, we offer recommendations for promoting co-creation in IS research.

### State of the literature on stakeholder collaboration during implementation

Successful design and deployment of implementation strategies typically require coordinated action among organizations, groups, and individuals. Participatory approaches used by IS researchers include implementation mapping (17–19), user-centered design (19), group prioritization processes (20), community advisory boards and expert panels (21–23). IS researchers have also adopted engagement approaches from community-based participatory research (CBPR) to enhance the active inclusion of all relevant stakeholders in IS work. CBPR necessitates shared leadership and co-learning relationships among researchers and community partners (24). CBPR elucidates the benefits of involving end-users in research planning and implementation of EBPs and other innovations, reducing stakeholder power differentials, and illuminating key factors to address health equity efforts (24).

Current collaborative approaches in IS lack concrete guidance on synergistically integrating all stakeholders' expertise, values, and priorities for the joint, integrated creation of knowledge. IS researchers would benefit from such guidance given variations in stakeholder backgrounds and lived experience, professional roles, access to resources (e.g., fiscal and material capital), and thus privilege and power represented at the table (3). We must better understand collaboration processes in IS that often unfold within complex contexts with stakeholders who may not share the same priorities. We must enhance our knowledge of how implementation collaborations operate in these contexts and ways to optimize them to benefit diverse stakeholders, including end-users who are often not included or meaningfully involved in collaborative processes.

## Consequences of “collaborations” lacking co-creation

Stakeholder engagement, governance arrangements, and building capacity for productive and successful co-creation can be challenging (14). Improved understanding of successful approaches for developing researcher and community stakeholder collaborations into co-creation partnerships is critical to achieving meaningful implementation and sustainment outcomes. Guidance on nurturing such collaborations should address power imbalances and support communication and trust among stakeholders (3). In co-creation relationships, all stakeholders ideally participate in and share control throughout all phases of research. This approach contrasts with traditional research dynamics that position researchers as external experts and gatekeepers of information. Such dynamics can perpetuate power differentials and information asymmetries between researchers and community stakeholders (25). Power imbalances may be heightened in research conducted with socially and economically marginalized communities (26). Power imbalances contribute to low acceptability, appropriateness and adoption of new practices, or abandonment of new practices soon after a study concludes (27, 28). Stakeholders lacking meaningful engagement in decision-making and implementation may be left without a clear understanding of how their participation contributes to results (29). Sustainment is challenged when stakeholders identify little value in or from their engagement in an implementation collaborative. Conversely, partnerships built on power-sharing and democratic principles can promote multilevel buy-in, capacity for change, and encourage adoption of new practices (30).

The absence of clear co-creation principles in IS fosters insufficient knowledge about individual actions and responsibilities for achieving implementation goals (31). Role ambiguity engenders confusion about what researchers or community partners are expected to do, curtailing their ability to improve or sustain EBP use (32). When researcher-provided resources recede at the end of the implementation phase, community stakeholders may struggle to organize necessary supports and sustain EBPs (32). Green et al. contend that “without significant changes, the adoption of co-production on its own will not lead to significant changes,” meaning we must become more intentional in overcoming challenges and applying knowledge from stakeholders (33).

These challenges may result in selecting EBPs that fit poorly with community needs, resources and priorities. Fit—*the perceived appropriateness of the intervention/implementation strategy and setting*—is central to implementation success (16). Researchers and community stakeholders must meaningfully consider their context when selecting an EBP, and be prepared to adapt interventions and strategies to accommodate for changing contextual influences (30, 34). Collaborations that support highly generalizable approaches to intervention design and testing may not fit well with local conditions, and thwart uptake (35).

## Proposed solution: Specifying practical and research applications of co-creation

Although IS underscores collaboration's value in implementation research, it lacks guidance for nurturing collaborative efforts, and ensuring they reflect contributions and meaningful participation from all stakeholders. Despite growing awareness that IS must proactively engage with health equity (4), IS researchers struggle with structuring equitable, collaborative processes to support transformative impacts through successful implementation. We draw from organizational research, community-engaged studies, and patient-centered care to argue that the concept of co-creation in implementation collaborations can catalyze contextually relevant insights and approaches to help reach expected outcomes. We next describe co-creation and the application of five co-creation principles through case examples.

### Concept of co-creation

Co-creation is the process of convening a diversity of stakeholders who are willing to share their knowledge, skillsets and resources to spark synergy and persevere to an end-result surpassing the sum of its parts (5, 6, 36). The goal is for these partners to contribute to the planning, design, testing, and implementation of the services they fund, deliver, or receive (37, 38). Although there is limited outcomes research on co-creation, current evidence suggest that co-creation leads to stakeholder trust, equitable contributions, and a sense of ownership (39), and in turn to quality research as well as meaningful research which meets individual and community expressed needs and goals (21). This concept is referred to as “co-design” or “co-production,” terms often used inter-changeably in the literature as they focus on jointly producing, designing or creating (e.g., knowledge to be applied, such as an intervention prototype or research design) (38, 40–44). We present co-creation as a multi-dimensional concept for “all things co” that necessitates meaningful engagement among stakeholders (45). This type of engagement requires co-creators (particularly researchers) to grapple with what it means “to open up their processes” to forge effective partnerships with different stakeholders (46). Members of ‘all things co' processes are often specified as stakeholders with relevant and unique expertise and experience to contribute. Graham et al. assert that participatory co-production processes are critical to advancing the science of evaluating stakeholder engagement (47, 48).

Much like CBPR, co-creation research is driven by power-sharing governance arrangements (e.g., partnership agreements) between stakeholders. It is guided by end-users who are *experts by experience* (49), meaning partners whose lived realities enable them to share knowledge, values, and needs that are often not known or fully appreciated by program developers or researchers. This type of exchange is often characterized as *local end user-driven* collaboration (50). Stakeholder voices and contributions are engaged at the behavioral, cognitive and/or emotional levels and shaped by the group's motivation(s) for collaborating (51).

Pearce et al. clarify that the co-creation of new knowledge for health interventions must address conceptual ambiguity and the pragmatics of participation by proposing core principles (rigorous research methods and embeddedness) (41). In a special issue of Evidence & Policy on co-creation, Metz [(32), p. 333] assert that the “legitimacy of co-creation approaches is underpinned by explicit core values and assumptions about how affected parties will be involved in the work.” To follow on this recommendation, we draw from a growing body of work (9, 25, 37–39, 52) to assert the following five principles of a co-created collaborative process to enhance implementation efforts:

1. **Equity:** This principle calls for greater equity in relationship-building among stakeholders, with end-user knowledge and experience being valued equally with that of professionals. By evoking equality, we do not naively assume each stakeholder holds equal power in collaboration. Rather all stakeholders in an implementation pursuit deserve *equitable access* to shared responsibility, decision-making power, and the resources required for participation. Equitable access recognizes that participation needs may differ across stakeholders based on individual (e.g., culture, preferences, and values), organizational (e.g., professional roles) and contextual characteristics of the implementation environment (e.g., resource-rich vs. underserved). Facilitators of co-creation group processes are tasked with promoting a more active role among implementers and end users in research (50). Equity is supported through access to information, networks and resources, transparency, and value alignment (9). Equity in relationships during co-creation engagement promotes trust and lead to meaningful engagement among non-academic partners and to higher engagement in the research process (53, 54).

Application in IS: This principle is applied by convening collaborations with multi-disciplinary academic researchers, implementers (e.g., service providers), end-users (e.g., patients, clients), and other relevant stakeholders (e.g., community leaders, policy makers) based on the nature of the effort (25). It is also reflected in re-designed governance structures before, during and after the implementation process and in stakeholders, especially researchers, striving to become more self-aware of implicit bias possibly affecting attitudes, interactions, and fundamentals (55).

**2. Reflexivity:** This principle acknowledges that researchers (and other co-creation partners) strive to be aware of and analyze how their positions within collaborative research processes may influence its dynamics, including how stakeholders interact with one another and engage in implementation (5, 56). Reflexivity is seen as critical to situating positionality and power within the collaborative, likely reducing stigma and promoting respect for all perspectives and values (57). This principle also supports sustainability and long-term goal setting as well as growth of partners' networks over time (57, 58).

Application in IS: Reflexivity requires making time and space for ongoing group reflections to identify and redress power imbalances and processes for sharing information and making decisions, and to recognize limitations of using dominant frameworks, including unintended consequences of well-intended research for diverse implementers and end-users, and social dynamics shaping our collaborations (57, 59, 60).

**3. Reciprocity & Mutuality:** This principle concerns the degree to which stakeholders are open and interested in learning from each other, referred as the “knowledge appetite” (50). Relationships are perceived and experienced as mutually beneficial through the combined and generative knowledge and the deepened connections and networks developed among all partners (50). This reciprocity leads to perceived stakeholder ownership of the collaboration process. This value can foster accountability, co-learning and learning transfer in a bi-directional fashion between researchers and other partners (32, 53).

Application in IS collaborations: Reciprocity and mutuality is promoted through the inclusion of stakeholders in power-sharing governance arrangements (36), and by researchers communicating the evidence base for potential implementation strategies to inform decision-making (25). Reciprocity can be achieved when co-creation stakeholders collectively create products useful to all partners including community-facing materials (e.g., toolkits, brochures) beyond research manuscripts.

**4. Transformative & Personalized:** This principle refers to a collaborative process that benefits the study while also offering an individual experience that is enriching, given the emphasis on end-user orientation through use-value and empathy (9). Research is perceived as having room for new possibilities because of the collaborative process. When this principle is met, it is easier to obtain buy-in and support from implementers (50). This principle can foster activation and self-advocacy among patients, families, and community members as a result of their works side-by-side with researchers (59). In addition, promoting an understanding of each partner's motivations for joining a collaborative, and opening the space for them to take on or lead roles that align with those motivations can become a transformative and meaningful participation for individuals (61).

Application in IS collaborations: This principle necessitates increasing knowledge and skills among non-academic stakeholders to relevant theory and research methods (25), and by organizing knowledge and skill-building activities during the Preparation phase, such as IS training boot camps. Training may also need to focus on increasing contextual knowledge and engagement skills for co-creation initiatives among researchers. Stakeholders are empowered to develop their own solutions (53) by participation in identifying and selecting interventions/implementation strategies and desired outcomes. These efforts create not only research value but also individual and community value.

**5. Relationships Facilitated:** Relationship structures (e.g., partnerships) or procedures (e.g., agreements on roles/responsibilities) are developed collectively to support a co-creation implementation collaborative. Participation is encouraged and facilitated through organizations and social networks and by creating explicit spaces and time for partners to network, invite their own networks to contribute to the implementation process at key phases of the project, and by formalizing roles and responsibilities in writing such as through Memorandum of Understanding or MOUs (50). Relationships are joint, reciprocal and fostered through iterative group processes, active communication, and/or engagement. Facilitated relationships promote trust, shared power, and problem-solving orientations necessary to sustain implementation efforts (61).

Application in IS: This principle is applied by structuring collaborations with diverse and inclusive implementation networks (which requires periodically reflecting on which stakeholders are not at the table and need to be (re)invited to participate), facilitating interdependence by engaging and using mutual resources across all stakeholders, and by building cooperative inter-organizational relationships through participation agreements (9).

### Application of principles through EPIS

A key recommendation for using IS frameworks is to establish and maintain community stakeholder partnerships (62). The co-creation concept fits well with existing frameworks. One prime example is EPIS, which encourages stakeholder engagement across the implementation ecosystem to facilitate efforts longitudinally and contextually (16, 63). For EPIS, co-creation is a bridging factor necessitating collaboration among stakeholders in the ecosystem's outer and inner contexts to shape an innovation's adoption and scale-up (64). Bridging factors are the relational ties, arrangements, and processes serving as the connective tissue across and between contexts (64).

**Figure 1:**
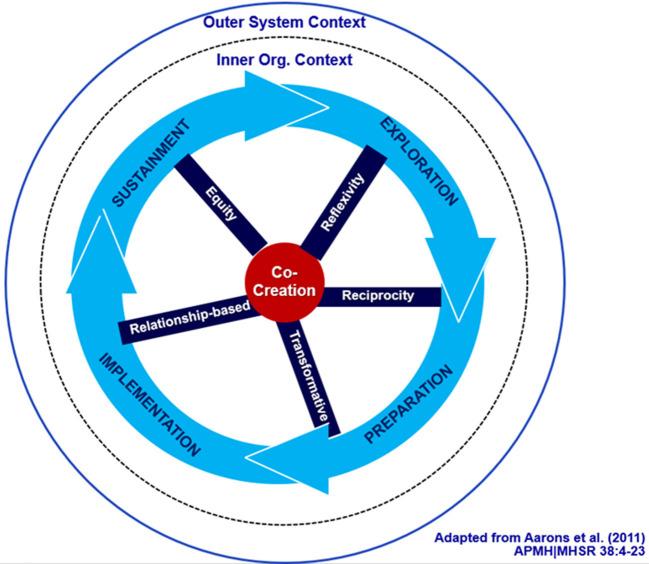
Co-creation EPIS model (65).

Co-creation principles can inform feedback-driven collaborations throughout EPIS phases to increase synergy and equity (Figure 1). Although the three case studies below all used the EPIS to guide the collaborative process, co-creation principles are transferable to other frameworks. An engagement process driven by co-creation principles compels us to critically look at power among partners and how it manifests across each of the EPIS phases. One example is the work of Stanton et al. (3) who offer critical questions to pose across the implementation phases as a way to more intentionally address power in implementation collaborations, and we would add co-creation principles.

A description of three federally-funded implementation studies are presented in the next section as case examples.

Example # 1: Participatory implementation approaches to advance health equity for gender diverse and sexual minority (GSM) students.

“Reducing LGBTQ+ Adolescent Suicide (RLAS),” or RLAS for short, is a cluster-randomized study that uses a multisectoral community-academic partnership (CAP) involving stakeholders from schools, intermediary organizations, state government, and research institutions. The trial operationalized EPIS using the Dynamic Adaptation Process (DAP) (66), a data-driven implementation planning methodology that was used to facilitate uptake of interventions to enhance school climates and reduce suicidal behaviors for GSM high school students in New Mexico. This inclusive planning methodology made it possible for the CAP to convene implementation resource teams of educators, health professionals, and youth in 19 high schools. As described below, the CAP-provided feedback and technical assistance. The teams engaged in iterative assessment and planning processes to build school capacity, and select and implement interventions/implementation strategies, working closely with researchers to co-create and deploy locally responsive educational materials, tools, and action plans to introduce inclusive practices in socially-conservative school communities.

Levels of partner engagement in RLAS spanned the modes of “involve,” “collaborate,” and “empower” on the Spectrum of Public Participation continuum (67). The process of context-driven adaptation and site-specific tailoring placed IRTs in the highest level of involvement (“empower”) because members were charged with all final decisions regarding implementation. While they were engaged throughout the study, the participation of CAP members ranged from the “involve” to the “collaborate” modes. National organizations were consulted on training materials and data collection, and provided critical information about outer-context efforts concerning school health and GSM advocacy. State agencies were similarly engaged, yet more directly involved in shaping study objectives, providing resources to school sites, and responding to and applying study findings.

They benefited directly from engagement with implementation sites, as RLAS allowed for increased access to school settings that were otherwise difficult to reach. National- and state-level members were more closely aligned with the “involve” mode in that their guidance influenced RLAS, as the core study team maintained continued dialogue with them throughout the course of their work. The members with a more intermediary function were closely aligned with the “collaborate” mode, in that they partnered closely with study coaches, IRTs, and schools to shape implementation on a local level.

Two primary, yet not unsurmountable, challenges affected participation in RLAS. First was staff turnover at all levels. For example, turnover in schools (e.g., constantly changing administration and IRT membership) could undermine progress in implementing GSM supportive practices. Although turnover in the IRTs exerted the greatest direct impact on implementation, personnel at intermediary organizations and state agencies also changed over this 5-year study. Time emerged as a second factor affecting IRTs specifically. As school staff were already stretched thin, time for IRT members to meet, plan, and carry out action items to support implementation came to represent a scarce, highly valuable resource.

Three main facilitating factors balanced the above challenges. First, coaches were key to establishing and maintaining connections among schools, study staff, and intermediary organizations, providing guidance to IRTs, recruiting new IRT members, obtaining administrative buy-in, and ushering resources from outer to inner contexts. Second, the team structure of IRTs and their ability to evolve according to the needs of schools was a boon to sustaining implementation progress despite changes in membership. Third, the personal and institutional relationships fostered through the CAP, coaches, and schools allowed for problem-solving, mutually beneficial leveraging of resources, and tailoring supports to school-based needs, including addressing challenges of time constraints and changes in staff.

Example # 2: Implementation mapping to co-create protocols for supporting state-mandated screening of children for Adverse Childhood Experiences (ACEs).

ACEs are potentially traumatic events occurring before age 18, such as maltreatment or exposure to violence (68, 69). ACEs screening identifies these events and their associated health and wellbeing outcomes. In 2020, California issued an “ACEs Aware” policy that reimburses primary care clinics for annual patient screenings. In partnership with a health system serving over 6,000 children annually, this randomized trial is testing the impact of ACEs screenings on child service access and outcomes as well as the role of a multi-faceted implementation strategy in supporting such screenings for children ages 0–5 years. The co-creation process involved: (a) the clinical partner bringing their identified service gap to academic partner (i.e., need to address patient trauma) to co-develop a plan of action, (b) establishing a Trauma-Informed Care (TIC). Workgroup comprised of clinical staff, providers and managers to address this gap during the Exploration phase; (c) bridging this gap by adopting the state's ACEs Aware policy; and (d) undertaking participatory implementation mapping (70) (i.e., six step iterative and systemic collaborative approach to develop, select and/or tailor multi-level implementation strategies) to co-create implementation strategies for screenings and protocols for delivering trauma-informed care for future pilot-testing. The type of engagement for this project, based on the Spectrum of Public Participation continuum, falls within the collaboration and empowerment levels. The researchers partnered with healthcare administrators, service providers, program managers, members of the Information Technology and Quality Improvement departments, and caregivers of child patients to make decisions on every phase of the research process. That is, partners were collaborators of researchers in 2019 during the exploration implementation phase when the healthcare system was considering and ultimately adopted the ACEs Aware program, in 2020 during grant proposal writing and in 2021–2022 once the project received federal funding, during the planning process and pilot testing of the implementation strategy.

In addition, partners were empowered to make final decisions on which challenges and aligned implementation strategies to focus on, how to structure the activities for implementation of the ACEs screenings, how to organize the planning groups in terms of structure and process, and to have the power to request changes to data collection timeline, as possible by the funding agency, to accommodate significant changes within their system (e.g., high turnover) as well as changes to the ACEs policy requirements or other external events (i.e., inner or outer contexts). During the engagement process, the identification of challenges was complemented with acknowledgment of facilitators or assets within the partner healthcare system. Main challenges faced by partners were the high turnover and the financial and personal impact of the COVID-19 pandemic (i.e., 2020–2022) for implementers, leadership and caregivers of child patients.

Departure from key partners significantly impact engagement and the co-creation process as their expertise, gained knowledge and experiences and support is lost during a period of time or permanently if the position is not filled as it happened often with our healthcare partners. Nonetheless, facilitators that were leveraged to inform strategies included the use of implementation mapping (70) that allowed partners to work within smaller workgroups during the planning phase. This engagement early on during exploration and preparation phases (16) facilitated partners' ability to fill in relatively quickly for colleagues no longer at the organization. In addition, having two co-leads (aka champions) who were internal healthcare personnel co-lead the project along with the research team starting during grant proposal writing facilitated troubleshooting, decision-making, and coordination.

Example # 3: CO-CREATE: co-creating a COVID-19 testing program to promote health equity in a U.S./Mexico border region.

CO-CREATE is a rapid response, mixed methods implementation research study funded by the National Institutes of Health Rapid Acceleration of Diagnostics for Underserved Populations (RADx-UP) initiative to co-design and implement a culturally responsive and competent COVID-19 testing program for San Diego communities near the U.S./Mexico border (71). Co-creation drives this community engagement project through several methods: (a) a Community Advisory Board of community health workers, healthcare providers and administrators, and public health researchers who developed a project-driving theory of change and engaged in Appreciative Inquiry, to evaluate selection and implementation of co-created COVID-19 public health strategies (71); (b) qualitative brainwriting data collection sessions with patients and providers to identify and address implementation barriers; (c) partnership and co-leadership of all project activities with a federally-qualified health center to promote successful implementation and refinement of the testing program. For this project, the level of partner engagement fell between the collaborate and empower modes on the Spectrum of Public Participation continuum. Specifically, through the Theory of Change process (71), the Community and Scientific Advisory Board members were invited to collaborate with community and academic organizing team to identify root causes of inequitable COVID-19 testing and to develop community-vetted solutions to mitigate these inequities. After completion of the Theory of Change, the Community and Scientific Advisory Board has been engaged in an Appreciative Inquiry process to guide the implementation and evaluation of the identified solutions from the Theory of Change (71).

A primary facilitator was the community partner leaders of the project, the Global Action Research Center, who are an intermediary non-profit organization with strong and enduring relationships with community-based and ethnically-based community organizations in the region. The Global Action Research Center identified and invited the community leaders and health workers who were members on the Community and Scientific Advisory Board. They also primarily led each meeting, which fostered trust among the Board members and with the academic research team that organized the project. Another facilitator was the ongoing and multi-method evaluation of engagement that the project team undertook. This included ethnography and survey measurement of partner engagement after each meeting that allowed for near real-time assessment to inform changes needed within Board meetings to promote equitable and meaningful engagement.

Balanced with these facilitators were two primary and naturally-occurring challenges. First, to ensure equitable participation of Board members who represented the Latino, Spanish-speaking communities that were prioritized for the project, the Community and Scientific Advisory Board was structured to host live Spanish language interpretation and translation at each meeting. While this incurred more costs and reserved time for interpretation and translation, this was critical for important community perspectives to be shared. Second, because the levels of engagement were within the collaborate and empower modes, this required significant resources in terms of person-hours and fiscal costs. An analysis of the community engagement resource needs and costs are reported elsewhere (71).

Table 1 provides an overview of how each of the EPIS phases can be approach through a co-creation lens. For each phase(s), we also provide an overview of the activities used by the case examples to meet each of the co-creation principles.

**Table 1:** Implementation research collaboration summary case analysis from a co-creation lens and guided by the EPIS framework.

	Co-Creation principle^*^	**Example 1: Health equity for gender diverse and sexual minority (GSM) students**	**Example 2: ACEs policy implementation in an FQHC system**	**Example 3: COVID-19 testing program at the US/Mexico border**
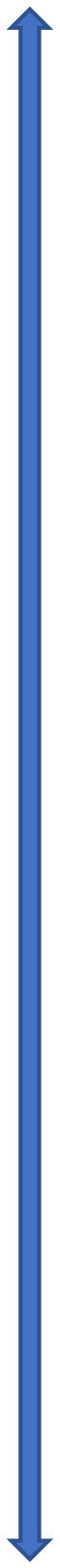	**EPIS exploration and preparation phases: From a co-creation lens, focus on understanding diversity, and power differentials in local contexts, examining who needs to be at the table, and agreeing on governance, roles, and responsibilities. Prioritize opportunities for interaction (in-person or virtual) to convene and work collaboratively with partners**			
	Equity in relationship building: End-user knowledge and experience being valued equally with that of professionals	• Convened a multidisciplinary and multisectoral CAB, workgroups, and IRTs • Subawards and monetary incentives for members and organizations to formalize roles and responsibilities	• Trauma Informed Care (TIC) workgroup members and study champions (FTE % covered) self-organized the healthcare system (clinics and central personnel) into implementation mapping workgroups • Bilingual community health advisors and Latinx mothers joined the planning process, honorarium for caregiver time	• Community partners and CAB members were identified via existing relationships • Compensation was provided to all co-creators *via* sub-awards or honoraria
	Reflexivity: Researchers (and other partners) strive to be aware of and analyze how their positions may influence the collaborative's dynamics	• Partners negotiated research design issues while sharing ideas informed by their unique but complementary positionalities to troubleshoot challenges and facilitators to implement GSM-centered school interventions	• Partners negotiated clinical efficiency of the screening process while accessing available resources • Planned composition of group meetings and separate follow-up meetings ensured that partners with traditional less power in clinics (e.g., staff, community health advisors, and caregivers) had additional spaces to share and fully participate	• After each CAB session, CAB members, community partners leading CAB sessions, and research team members completed a validated survey to assess partner engagement
	Reciprocity & mutuality: Partners are interested in learning from each other. Relationships are perceived and experienced as mutually beneficial through the combined knowledge and the deepened networks developed	• Partners recognized and reinforced their shared commitment to reducing health disparities for GSM youth through consistent participation and by celebrating successes both large and small, particularly the co-design and sharing of training materials	• Power-sharing governance was structured by funding a percentage of clinical staff salaries and including them in weekly research meetings • Discussions of benefits and unintended consequences resulted in several concrete actions such as adding a strength-based section, focused on family resiliency, to the REDCap screening system	• Benefit was assessed through willingness to collaborate on projects, products, proposals beyond the current project and through ethnographic and survey assessment of CAB member engagement satisfaction and benefit to their personal and professional work
	Transformative & personalized: The collaborative process benefits the study while also offering an enriching individual experience through use-value and empathy	• Qualitative interviews with partners, young people with lived experience, and technical assistance providers pointed to the value of the engagement experience, with one partner coming out of retirement to promote GSM student health, another changing their academic career path to focus on GSM student health, and a third securing employment at a large school district to implement programs to improve school climate and culture for GSM students	• Members of the healthcare system identified the need for TIC training for their clinic staff to address burnout and self-care and to focus on a culturally relevant approach to screenings • National coaches (one coach was bicultural and bilingual Spanish) provided this training to clinics based on their identified need	• Primarily assessed through CAB evaluation methods that include both ethnographic assessment in CAB sessions and a self-report survey completed by CAB members using the Goodman et al. (72) engagement survey
	Relationships facilitated: Relationship structures and procedures are developed collectively to support the implementation collaborative	• Dialogue among school nurses, school health advocates, and academic partners set the foundation for the CAB • The structure of the collaboration shifted to address needs (e.g., workgroups to develop professional education competencies)	• A multilevel network structure was developed: (a) TIC workgroup meeting monthly/quarterly, (b) bi-monthly meetings with top executives, (c) planning sub-groups in five areas of development, and (d) caregiver group as advisors during the planning process	• The CAB intentionally includes a mix of community health workers, clinical providers and staff, and researchers
	**EPIS implementation phase: From a co-creation lens, focus on deepening partner relationships, and monitoring the collaborative's activities to make sure they are meeting relevant co-creation principles or goals; focus on addressing each partner needs to maintain collaborative capacity**			
	Equity in relationship building: End-user knowledge and experience being valued equally with that of professionals	• Partners expanded to include new youth liaisons, intermediary organizations, school, and state agency personnel with situated knowledge and expertise, and were resourced as needed to contribute to project activities	• Use of coaching and feedback with community health advisors once or twice a month to troubleshoot and listen to their suggestions for adaptations	• Simultaneous Spanish translation promoted equitable access, and information sharing during CAB meetings
	Reflexivity: Researchers (and other partners) strive to be aware of and analyze how their positions may influence the collaborative's dynamics	• Formal periodic reflections with study coaches and community-based technical assistance experts enhanced partner understanding of challenges and potential solutions • The contributions of partners were tracked, including for the co-design and delivery of local, state, and national presentations • These contributions were formally acknowledged for their influence on engagement and implementation efforts	• The composition of implementation mapping sub-groups was revised to add members or move members to a different group based on their role/expertise and preference • The TIC workgroup served as a space in which to discuss potential care team members' burnout and emotional stress due to ongoing ACEs conversations with caregivers	• Community members suggested that Spanish-speaking members be invited to speak first or in more explicit ways to encourage more equitable participation • Partner engagement surveys included items on shared power in decision-making, and open-ended questions to solicit critiques of and recommendations for engagement
	Reciprocity & mutuality: Partners are interested in learning from each other. Relationships are perceived and experienced as mutually beneficial through the combined knowledge and the deepened networks developed	• Non-academic partners forged or further cultivated mutually beneficial connections in the broader collaboration, resulting in new and stronger initiatives to address GSM student health • Non-academic partners advocated and raised awareness of key outer-context determinants (e.g., legislation) to leverage to enhance health equity for GSM students in schools and statewide	• The project's clinical co-lead received introductory training in Implementation Science provided nationally (i.e., Implementation 101) • Academic partners learned about potential unintended consequences of ACEs screenings and the impact of COVID-19 on the capacity to innovate within the partnered clinical system	• New opportunities for collaboration among academic and non-academic partners were shared, resulting in several new proposals and dissemination products
	Transformative & personalized: The collaborative process benefits the study while also offering an enriching individual experience through use-value and empathy	• Regular check-ins with partners (including IRT members) ensured the timely identification of needs and facilitated involvement in project activities through equitable engagement • Partners agreed on the collective value that GSM student health is a major societal health issue that can no longer be neglected, and that collaboration is the way to prioritize and address this issue	• Partners regularly implemented an adapted co-creation survey (9) to assess how partners perceived the individual value of their participation in the ACEs implementation collaborative • Partners strove to build implementation capacity through ongoing training and coaching	• The review of survey results after every CAB session informed ways to modify group processes to promote equitable engagement, such as encouraging non-academic partners to share their perspectives first • Continuous assessment of values alignment across within multilevel partnerships
	Relationships facilitated: Relationship structures and procedures are developed collectively to support the implementation collaborative	• An annual training institute was co-created to develop skills and intentionally nurture mutually supportive relationships among the IRTs and other partners, affording time and space to individually and collectively reflect on lessons learned and encourage each other's implementation efforts • Resources (e.g., coordination and communication support) were key to maintaining structures for co-creation	• A multilevel group structure (i.e., management, quality department, IT, providers, health advisors, and patients) derived from the implementation mapping workgroups facilitated the continuation of partner engagement during extreme turnover due to the COVID-19 pandemic • Iterative communication flowed upwards to clinic executives and other leaders and downwards to staff and caregivers	• Monthly CAB meetings provided a socially safe space that, over time, led to increased comfort in sharing personal experiences and trust among the partners

* Abbreviated definitions due to space limitations. ACEs, Adverse childhood experiences; CAB, Community advisory board; GSM, Gender and sexual minority; IRT, Implementation resource teams; IT, Information technology; TIC, Trauma-Informed care.

#### EPIS sustainment phase

From a co-creation lens, the focus should be on supporting partners to lead their own engagement process locally to maintain goals achieved and to continue the implementation collaborative, if relevant. This work entails the exploration of alternative funding opportunities and new partnerships based on the shared commitment to addressing emergent and dynamic needs. Some of the case example projects described here are further along in the Sustainment Phase than others. All projects are currently seeking to maintain funding and developing new or complementary projects with partners. Connections developed in previous EPIS phases have allowed for continued resource provision after the withdrawal of study support. These connections also serve as a springboard for co-designing new initiatives. During this phase, attention to dissemination practices that adhere to each co-creation principles as a guide (e.g., sharing with equity, reciprocity, and mutuality) may involve concrete activities, including efforts to gradually shift control and decision-making to local champions through implementation coaching and feedback, co-presentations at academic and community forums, and new training opportunities for partners grounded in emerging needs and priorities from the co-created implementation process.

## Discussion

In this commentary, we argue for co-creation in IS collaborations using five principles and by linking IS activities linked to each principle. These principles are transferable to any research area to enable a synergistic collaborative process. They can also foster longer-term relationships that can support resource intensive implementation efforts and sustainment of new practices. Thus, it is critical for researchers, implementers, and community partners to engage in co-creation to identify the need for change, the research-practice gap to address, prepare for and implement new practices, and sustain efforts long-term. The rich and inter-dependent knowledge that a co-created process promotes across diverse stakeholders is critical for ensuring fit and relevance to local contexts.

We recognize the challenges to co-creation in IS, with available infrastructure and time varying dramatically among academics, funders, and community-based stakeholders. Co-creation should not be expected to be a tidy process—it requires time, compromise and means IS researchers might need to step outside their comfort zones. We need to embrace rather than eschew the tensions possibly arising through co-creation (38), as they likely comprise a source of creativity and new ideas to plan for successful implementation. Furthermore, there is also a need for researchers to be clear on what exactly needs to be co-created and to balance a co-created process with the expectations of rigorous scientific endeavors (73). Co-creation in IS will require re-evaluating prioritization of academic knowledge and frameworks that do not align with or are irrelevant for community partners, especially given cultural, language, and social differences. Last, promoting implementation co-created implementation collaborations require specific knowledge and skills need to be incorporated into existing IS training.

In this paper, we make a case for further developing the concept of co-creation in IS with the goal of answering an ultimate question:

*Does a co-created implementation collaboration provide stakeholders, especially end-users and community partners, with a deepened capacity to advocate for quality services, and as defined by local communities?*

IS researchers may be familiar with system-level challenges to collaboration, but less aware of dynamics specific to local contexts. This blind spot can compromise their understanding of barriers, facilitators, mechanisms of change associated with implementation. Co-creation will enable such insights, enhancing the scientific value of our evaluations, our ability to replicate outcomes, and increasing the potential for achieving health equity and social justice through successful implementation of needed interventions.

In this paper, we present co-creation as a multidimensional concept and identified five concrete principle that were illustrated through three case examples. These principles have pragmatic values as they can be transferable across groups, topics and systems. Implementation collaboratives can use a prioritization approach to selecting all or the relevant co-creation principles, and as standard goals for the group. Then, partners can identify concrete activities that will allow them to achieve each of those goals and that align with the EPIS phases as presented in Table 1. This mapping of standard goals and tailored activities can facilitate ongoing monitoring and evaluation of the co-creation process through rapid iterative cycles (74). We also recommend complementing this approach by raising Stanton et al. (3) power-based questions for each of the EPIS implementation phases and as a way to translate co-creation from a high face validity value in engagement research to a formal and more standard practice in implementation research.

Prior research have used qualitative interviewing and ethnographic approaches to describe how co-creation builds co-creative relationships that support ongoing collaboration and problem-solving to sustain and scale out implementation efforts (53, 58, 61). However, there is still a dearth of outcomes research on co-creation (32). Mixed methods research is needed to simultaneously measure and explain the impact of co-creation on implementation proximal and distant outcomes such as partner experience, adoption, appropriateness, feasibility and sustainment. Last, future research should explore the alignment of co-creation with spectrums of engagement such as the IAP2 model (67). From that model, if collaboration and empowerment are seen as suitable levels of engagement to be achieved by a group, co-creation could be the vehicle to reach that goal.

An IS approach foregrounded in co-creation will help us better elucidate aspects of collaborations that adhere to co-creation principles, and whether outcomes are achieved through synergistic and equitable approaches among diverse stakeholders. It is our aspirational goal that the co-creation principles we described will inform current efforts to assess the quality of co-produced research (71), and that they will become a more normative and explicit application in IS research.

## Author's note

There is growing interest for the field of implementation science research to promote the active engagement of diverse and often disenfranchised stakeholder voices. Including multiple perspectives can increase project buy-in, promote adherence to implementation protocols, improve the selection of meaningful outcomes, and overall sustainment of evidence-based practices. Currently, there is no clear guidance to help implementation science (IS) researchers identify and build collaborative approaches guided by co-creation principles for working with the community. This manuscript provides background on IS collaborations and identify the following co-creation principles relevant to the field and informed by organization, community-engaged and patient-centered literatures: (1) equity, (2) reflexivity, (3) reciprocity and mutuality, (4) transformative and personalized collaborative processes, and (5) relationships facilitated through inclusive networks, iterative processes, and active communication. Applications of these principles are further described using three IS research case examples. Principles for structuring co-creation collaborations will help researchers and community members engaged in implementation science research to spark synergy, address imbalances in power, privilege, knowledge, and access to resources. These principles will help researchers and community members collaborate in meaningful, equitable ways. Additionally, these principles provide the field with guidance that can be tested for its impact on producing meaningful, co-creation collaborations.

## Data availability statement

The original contributions presented in the study are included in the article/supplementary material, further inquiries can be directed to the corresponding author.

## Author contributions

MP conceptualized the original idea for this manuscript and led the manuscript drafting and revision process. All authors drafted text, contributed original ideas across different sections of the manuscript, revised, and approved of the final manuscript text.

## Funding

This work was partially supported by the National Institute of Mental Health R21MH123835 (MP); National Institute of Child Health and Human Development (R01HD083399; CW); NIMH K23 MH110602 (NS); R34 MH120190 (NS); P42 ES010337-19S2 RADx-UP Supplement (NS). JH works at the Center for Development, Evaluation, Complexity, and Implementation in Public Health Improvement (DECIPHer) which was funded by Welsh Government through Health and Care Research Wales; K01 DA056838 (EC); R01 DA049891 (EC and GA); P50 MH113662 (RL-H); P50 MH126231 (NS and GA). EC and RL-H are fellows, MP and NS are alumni, and GA is core faculty with the Implementation Research Institute (R25 MH080916). EC is also a fellow with the Lifespan/Brown Criminal Justice Research Training Program (R25 DA037190).

## Conflict of interest

The authors declare that the research was conducted in the absence of any commercial or financial relationships that could be construed as a potential conflict of interest.

## Publisher's note

All claims expressed in this article are solely those of the authors and do not necessarily represent those of their affiliated organizations, or those of the publisher, the editors and the reviewers. Any product that may be evaluated in this article, or claim that may be made by its manufacturer, is not guaranteed or endorsed by the publisher.
